# Supplementary material for: Effect of adaptive cruise control on fuel consumption in real-world driving conditions
Source: Nat Commun. 2024 Nov 19;15:10016. doi: 10.1038/s41467-024-54066-8 (PMC11577072; doi:10.1038/s41467-024-54066-8)
Supplement: Supplementary file 1 — Supplementary Information [file 41467_2024_54066_MOESM1_ESM.pdf]

Supplementary Information: Effect of Adaptive Cruise Control on Fuel Consumption in Real-World Driving Conditions

Ayman Moawad[1,*], Matthew Zebiak[2], Jihun Han[1], Dominik Karbowski[1], Yaozhong Zhang[1], Aymeric Rousseau[1]

[1]A. Moawad, J. Han, D. Karbowski, Y. Zhang, and A. Rousseau are with the Vehicle and Mobility Simulation department at Argonne National Laboratory, 9700 S Cass Ave, Lemont, IL 60439 USA.
[2]M. Zebiak is a Senior Consultant at 2050 Partners, 81 Coral Drive, Orinda, CA 94563.
[*]Corresponding author: Ayman Moawad (E-mail: amoawad@anl.gov)

Supplementary Table 1: Linear mixed effect model estimates of the effect of ACC engagement and other covariates on fuel consumption over various situation modes with preceding vehicle (PV) differentiation. The main numbers represent the estimated effects evaluated using t-tests based on Satterthwaite's method; numbers in parentheses represent the standard errors, and reported p-values are two-sided based.

	Dependent variable					
	FuelCons					
	(1)	(2)	(3)	(4)	(5)	(6)
	CrsPV	BSnAPV	BnAPV	APV	BPV	CrpPV
Inverse Avg Speed $(km/h)^{-1}$	5.287***	2.869***	2.180***	4.408***	3.348***	2.800***
(veh_spd_meanI)	(0.278)	(0.181)	(0.325)	(0.258)	(0.212)	(0.197)
ACC Engagement	0.500***	0.498*	0.575***	0.431	0.190	−1.066
(ACC_engaged_cat)	(0.054)	(0.276)	(0.092)	(0.268)	(0.119)	(2.033)
Vehicle Present Ahead	−0.443***	−0.617***	−0.505***	−0.609***	−0.723***	−0.228
(veh_ahead_cat)	(0.025)	(0.097)	(0.036)	(0.114)	(0.051)	(1.833)
Inverse Trip Distance (1/km)	0.310***	3.058***	2.500***	2.732***	−0.205***	0.855***
(dist_covered_kmI)	(0.006)	(0.026)	(0.014)	(0.031)	(0.014)	(0.254)
Starting Vehicle Speed (km/h)	−0.123***	−0.143***	−0.124***	0.472***	−1.500***	−0.049
(veh_spd_start)	(0.003)	(0.002)	(0.001)	(0.076)	(0.105)	(0.090)
Ending Vehicle Speed (km/h)	0.150***	0.260***	0.236***	−1.590***	0.593***	0.168**
(veh_spd_end)	(0.003)	(0.003)	(0.001)	(0.159)	(0.027)	(0.085)
Min Vehicle Speed (km/h)	−0.077***	−0.164	−0.117***	−0.687***	−0.570***	
(veh_spd_min)	(0.003)	(0.404)	(0.001)	(0.076)	(0.027)	
Max Vehicle Speed (km/h)	0.135***	0.149***	0.099***	1.988***	1.480***	−0.089
(veh_spd_max)	(0.003)	(0.004)	(0.002)	(0.159)	(0.105)	(0.157)
Trip Elevation Change	0.033***	0.296***	0.236***	0.383***	0.267***	0.363*
(elev_delta)	(0.0004)	(0.006)	(0.002)	(0.007)	(0.004)	(0.188)
Avg. Ambient Temperature	0.011***	0.152***	0.050***	0.073***	0.030***	0.565***
(amb_temp)	(0.001)	(0.005)	(0.002)	(0.006)	(0.003)	(0.068)
Avg. Engine Temperature	−0.044***	−0.109***	−0.077***	−0.108***	−0.022***	−0.362***
(eng_temp)	(0.001)	(0.002)	(0.001)	(0.003)	(0.002)	(0.047)
Vehicle Ahead While ACC Engaged	−0.516***	−1.077***	−1.005***	−1.294***	0.228*	
(ACC_engaged_catTRUE:veh_ahead_cat)	(0.059)	(0.290)	(0.100)	(0.295)	(0.128)	
Constant	−0.094	−5.334***	0.390	−7.488***	4.028***	27.731***
	(0.212)	(0.681)	(0.402)	(0.710)	(0.270)	(6.050)
Observations	92,434	38,580	81,661	13,033	26,992	351
Log Likelihood	−235,737.600	−126,204.500	−234,452.200	−40,045.500	−71,789.090	−1,347.710
Akaike Inf. Crit.	471,511.300	252,445.100	468,940.400	80,127.000	143,614.200	2,727.421
Bayesian Inf. Crit.	471,681.100	252,599.200	469,108.000	80,261.550	143,761.800	2,789.194

Note: *p<0.1; **p<0.05; ***p<0.01

Supplementary Table 2: Vehicles in the fleet. Unique make/model/series/trim combinations are distributed over multiple drivers and VINs, bringing the fleet to 157 vehicles on the road. Engine characteristics are as follows. LSY: 2.0T I4 Gas, L3B: 2.7T I4 Gas, LGY: 3.0TT V6 Gas, L84: 5.3L V8 Gas, L87: 6.2L V8 Gas, LT4: 6.2L Supercharged V8 Gas, LM2: 3.0T I6 Diesel, LZ0: 3.0T I6 Diesel.

Model Year	Make	Model	Series	Engine Model
**2020**	CADILLAC	CT5	Null	LSY
**2021**	CADILLAC	CT4	V-Series	L3B
		CT5	Premium Luxury	LGY
			V-Series	LGY
		Escalade	Premium Luxury	L87
			Premium Luxury Platinum	L87
				LM2
			Sport	L87
				LM2
			Sport Platinum	L87
				LM2
		Escalade ESV	Premium Luxury	L87
			Premium Luxury Platinum	L87
				LM2
			Sport	L87
			Sport Platinum	L87
	CHEVROLET	Suburban	High Country	L87
		Tahoe	LS	L84
	GMC	Yukon	SLT	L84
		Yukon XL	XL SLT	L84
**2022**	CADILLAC	CT4	Premium Luxury	L3B
				LSY
			Sport	LSY
			V-Series	L3B
		CT5	Premium Luxury	LGY
				LSY
			Sport	LSY
			V-Series	LGY
		Escalade	Premium Luxury	L87
			Premium Luxury Platinum	L87
			Sport	L87
				LM2
			Sport Platinum	L87
		Escalade ESV	Premium Luxury	L87
				LM2
			Premium Luxury Platinum	L87
				LM2
			Sport	LM2
			Sport Platinum	L87
				LM2
	CHEVROLET	Silverado	1500	L84
				L87
				LM2
		Tahoe	High Country	L87
	GMC	HUMMER	3X EDITION 1	Null
		Sierra	1500	L84
				L87
				LM2
		Yukon	AT4	L84
**2023**	CADILLAC	Escalade	V-Sport	LT4
		Escalade ESV	V-Sport	LT4
	CHEVROLET	Silverado	1500	LZ0
	GMC	Sierra	1500	LZ0
